# Potent and selective small-molecule MCL-1 inhibitors demonstrate on-target cancer cell killing activity as single agents and in combination with ABT-263 (navitoclax)

**DOI:** 10.1038/cddis.2014.561

**Published:** 2015-01-15

**Authors:** J D Leverson, H Zhang, J Chen, S K Tahir, D C Phillips, J Xue, P Nimmer, S Jin, M Smith, Y Xiao, P Kovar, A Tanaka, M Bruncko, G S Sheppard, L Wang, S Gierke, L Kategaya, D J Anderson, C Wong, J Eastham-Anderson, M J C Ludlam, D Sampath, W J Fairbrother, I Wertz, S H Rosenberg, C Tse, S W Elmore, A J Souers

**Affiliations:** 1 Oncology Development, AbbVie, Inc., 1 North Waukegan Road, North Chicago, IL 60064, USA; 2 Genentech, Inc., 1 DNA Way, South San Francisco, CA 94080, USA

## Abstract

The anti-apoptotic protein MCL-1 is a key regulator of cancer cell survival and a known resistance factor for small-molecule BCL-2 family inhibitors such as ABT-263 (navitoclax), making it an attractive therapeutic target. However, directly inhibiting this target requires the disruption of high-affinity protein–protein interactions, and therefore designing small molecules potent enough to inhibit MCL-1 in cells has proven extremely challenging. Here, we describe a series of indole-2-carboxylic acids, exemplified by the compound A-1210477, that bind to MCL-1 selectively and with sufficient affinity to disrupt MCL-1–BIM complexes in living cells. A-1210477 induces the hallmarks of intrinsic apoptosis and demonstrates single agent killing of multiple myeloma and non-small cell lung cancer cell lines demonstrated to be MCL-1 dependent by BH3 profiling or siRNA rescue experiments. As predicted, A-1210477 synergizes with the BCL-2/BCL-X_L_ inhibitor navitoclax to kill a variety of cancer cell lines. This work represents the first description of small-molecule MCL-1 inhibitors with sufficient potency to induce clear on-target cellular activity. It also demonstrates the utility of these molecules as chemical tools for dissecting the basic biology of MCL-1 and the promise of small-molecule MCL-1 inhibitors as potential therapeutics for the treatment of cancer.

Anti-apoptotic proteins such as BCL-2, BCL-X_L_ and MCL-1 maintain cell survival by binding and sequestering their pro-apoptotic counterparts, such as BAK, BAX, or the BCL-2 homology 3 (BH3)-only proteins BAD and BIM.^1, 2, 3^ Because cancer cells must survive amidst a variety of environmental stresses, they often express high basal levels of these BCL-2 family complexes and have been described to be ‘primed for death'.^4^ For the past two decades, teams of basic and translational scientists have worked to generate small-molecule inhibitors of these protein–protein interactions, with the aim of driving cancer cells to initiate apoptosis.

Although drugging these interactions has proven particularly challenging, intensive structure-based efforts have enabled the design of potent and cell-active BCL-2 family inhibitors. ABT-737 was among the first molecules described,^5^ followed soon thereafter by an orally bioavailable molecule, ABT-263 (navitoclax).^6^ Both molecules mimic BAD, with high affinity for BCL-2, BCL-X_L_ and BCL-W, and both molecules have demonstrated impressive anti-tumor activity preclinically.^7, 8^ Although navitoclax also demonstrated promising signs of clinical activity, its development has been complicated by dose-limiting thrombocytopenia, the result of BCL-X_L_ inhibition.^9, 10^ This prompted the development of ABT-199/GDC-0199 (venetoclax), a BCL-2-selective inhibitor that maintains anti-tumor efficacy while sparing platelets.^11^ Selective BCL-X_L_ inhibitors have also been generated^12, 13, 14, 15^ and the most potent molecules A-1155463 and A-1331852 demonstrate significant anti-tumor effects alone or in combination with chemotherapeutics (manuscript submitted).

None of the BCL-2 family inhibitors described above can inhibit MCL-1, and hence, not surprisingly, this protein has emerged as a potential resistance factor for these agents.^16, 17, 18, 19^ MCL-1 has also been implicated in mediating resistance to a variety of commonly used chemotherapeutic agents,^20, 21, 22^ and so generating small molecules capable of inhibiting MCL-1 represents an attractive approach for circumventing drug resistance. MCL-1 is a compelling cancer target in its own right, having been implicated in mediating the survival of multiple tumor types.^23^ The *MCL1* gene locus is amplified in a variety of tumor types, including breast cancer and non-small cell lung cancer (NSCLC),^24^ and the MCL-1 protein has been shown to mediate survival in models of multiple myeloma,^25, 26^ acute myeloid leukemia^27^ and NSCLC^28, 29^ and MYC-driven lymphomas.^30^

A variety of approaches for inhibiting MCL-1 have been described, including the use of BH3 peptides^31, 32, 33, 34^ and small molecules^35, 36, 37, 38, 39^ that bind MCL-1 directly or inhibit its expression indirectly.^18, 40, 41, 42^ Of the direct small-molecule inhibitors reported, none possess MCL-1 affinity within a range that would be expected to confer on-target cellular effects. Indirect MCL-1 inhibitors include cyclin-dependent kinase inhibitors such as roscovitine, flavopiridol, seliciclib, dinaciclib, and SNS-032, which inhibit the phosphorylation of the RNA polymerase 2 C-terminal domain and the elongation of transcripts, including *MCL1*.^40, 41, 42^ Because of its short (approximately 30 min) half-life, MCL-1 protein is rapidly eliminated upon treatment with flavopiridol or dinaciclib.^40^ Anthracyclines such as daunorubicin have also been shown to repress MCL-1 expression.^43^ A potential liability of the indirect MCL-1 inhibitors is that they also reduce the expression of numerous other short-lived proteins, making them less selective and potentially more toxic.

Here we introduce a series of direct, potent and selective MCL-1 inhibitors that demonstrate clear on-target cellular activity, disrupting MCL-1–BIM protein complexes and triggering apoptosis in cancer cell lines shown to rely on MCL-1 for survival. As expected, these molecules sensitize a variety of tumor cell lines to the BCL-2/BCL-X_L_ inhibitor navitoclax. To our knowledge, these molecules represent the first direct inhibitors of MCL-1 with the potency and properties required to inhibit MCL-1 in living cells.

## Results

### A-1210477 binds to MCL-1 with high affinity and induces MCL-1 protein elevation in cells

Recently, we described the synthesis of small-molecule inhibitors of MCL-1 elaborated from an indole-2-carboxylic acid core.^44^ Although molecules with the same core were also described by another group,^38^ those molecules were less elaborated, had lower affinity (*K*_i_>0.050 *μ*M) for MCL-1, and were not reported to demonstrate cellular activity. A-1210477 (Supplementary Figure S1) is a particularly strong binder of MCL-1 (*K*_i_=0.000454 *μ*M in time-resolved fluorescence resonance energy transfer (TR-FRET)-binding assays), representing an affinity improvement of at least two orders of magnitude over the aforementioned molecules, but is a much weaker binder of BCL-2 (*K*_i_=0.132 *μ*M) and BCL-X_L_ (*K*_i_>0.660 *μ*M). A-1155905, A-1208746, and A-1248767 are closely related analogs with similar affinity profiles (Supplementary Figure S1 and Supplementary Table S1).

**Figure 1:**
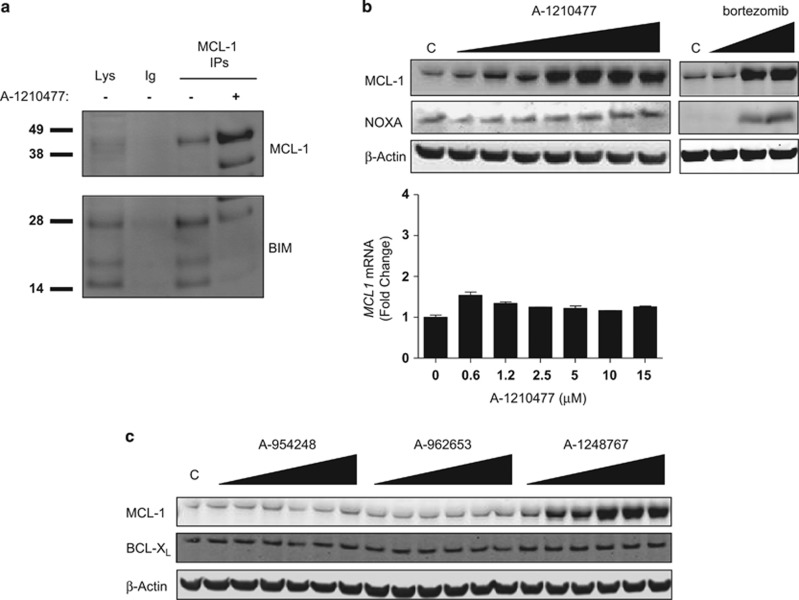
MCL-1 levels are elevated in cancer cells after treatment with MCL-1-binding small molecules. (**a**) H929 multiple myeloma cells were treated for 4 h with 10 *μ*M A-1210477 before immunopreciptating MCL-1. Immunoprecipitates were then analyzed by immunoblotting for MCL-1 and associated BIM. A control H929 lysate was run in lane 1, and an immunoprecipitation with a non-specific antibody (Ig) was run in lane 2. The positions of molecular weight markers (specified in kiloDaltons) are indicated on the left. (**b**) HCC-1806 breast cancer cells were incubated with increasing concentrations of A-1210477 (0.1, 0.5, 1, 5, 10, 15, or 20 *μ*M) or bortezomib (2, 10, or 50 nM) for 8 h before assessing MCL-1 and NOXA levels by immunoblotting. *β*-Actin was probed as a protein loading control. The lower graph depicts reverse transcriptase-PCR quantification of *MCL1* mRNA from HCC-1806 cells treated for 8 h with similar concentrations of A-1210477. (**c**) HCC-1806 cells were incubated with increasing concentrations (0.1, 0.5, 1, 5, 10, and 20 *μ*M) of A-954248, A-962653, or A-1248767 for 24 h before assessing MCL-1 and BCL-X_L_ levels by immunoblotting. *β*-Actin was probed as a protein loading control

As a first test of the potential cellular activity of these molecules, we assessed their ability to compete with the BH3-only protein BIM for binding to MCL-1 in H929 cells using co-immunoprecipitation assays. Four hours of treatment with 10 *μ*M A-1210477 were sufficient to reduce the amount of BIM co-immunoprecipitated with MCL-1 antibody, even though significantly more MCL-1 was pulled down from A-1210477-treated H929 cells relative to vehicle-treated controls (Figure 1a). Upon investigating the latter effect, we found that A-1210477 triggered MCL-1 elevation in a variety of cancer cell lines, including the breast cancer cell line HCC-1806 (Figure 1b). In contrast to the proteasome inhibitor bortezomib, A-1210477 had no effect on the levels of the MCL-1-binding protein NOXA. No corresponding increase in MCL1 mRNA was observed within the same time frame, indicating that this was not a transcriptional response. The A-1210477 analog A-1248767 induced similar concentration-dependent elevations in MCL-1 protein but not in BCL-X_L_ (Figure 1c). Increases in MCL-1 protein could be observed at concentrations as low as 0.5 *μ*M for A-1210477 and A-1248767, which have MCL-1 affinity constants in the range of 0.0004–0.0005 *μ*M (Figures 1b and c; Supplementary Table S1), whereas less elaborated compounds like A-954248 (K_i_=0.348 *μ*M) and A-962653 (K_i_=0.130 *μ*M) had no effect on MCL-1 levels up to concentrations as high as 20 *μ*M (Figure 1c). Intriguingly, the exogenous expression of known MCL-1-binding proteins such as PUMA and BIM2A has been shown to cause similar elevations of MCL-1 protein, likely by precluding the binding of BH3-only proteins such as NOXA and MULE, which are known to mediate the ubiquitylation and proteasomal degradation of MCL-1.^45, 46^ Hence, the data shown here provide the first indication that these small molecules are acting as BH3 mimetics that directly target MCL-1.

### A-1210477 disrupts MCL-1–BIM complexes in live cells

**Figure 2:**
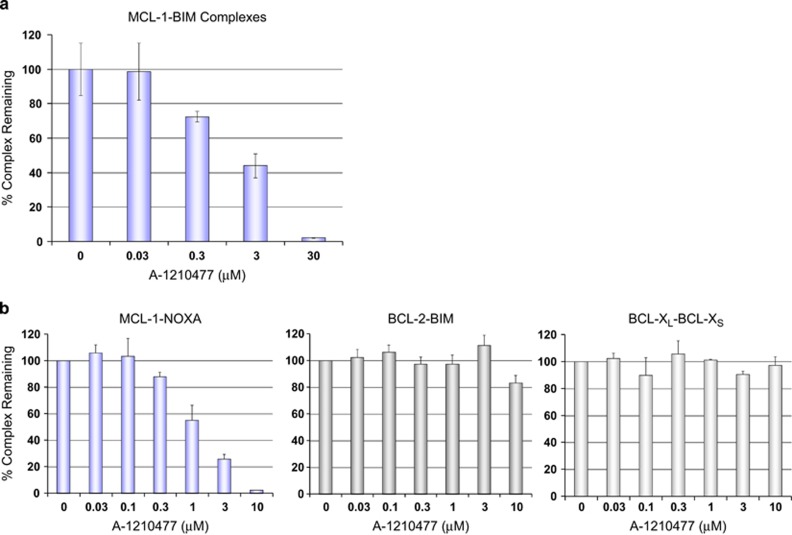
MCL-1 inhibitor A-1210477 disrupts of MCL-1–BIM complexes. (**a**) H929 cells were treated with increasing concentrations of A-1210477 for 4 h. MCL-1 antibody was used to capture MCL-1 and the associated BIM, which was detected using a MesoScale electrochemiluminescence assay. All data represent the mean of triplicate experiments, with error bars indicating the S.D. (**b**) Quantitative measurement of protein–protein interactions using a luciferase-based mammalian two-hybrid assay. HeLa cells stably transfected with a GAL4-driven luciferase reporter and expressing paired GAL4DBD–MCL-1:VP16AD–NOXA, VP16AD–BCL-2:GAL4DBD–BIM or GAL4DBD–BCL-X_L_:VP16AD–BCL-X_S_ fusion proteins were incubated with increasing concentrations of A-1210477 for 24 h. All data represent the means of triplicate experiments, with error bars indicating the S.Ds.

The observation that A-1210477 and A-1248767 behaved akin to BH3-only proteins in elevating MCL-1 levels indicated that these compounds are cell permeable and bind MCL-1 directly. To confirm this, we implemented a variety of orthogonal approaches to measure the effects of these compounds on cellular MCL-1–BIM complexes. We first employed a coupled co-immunoprecipitation–enzyme-linked immunosorbent assay (ELISA) protocol, beginning with immunoprecipitation and plate-based capture of MCL-1 and its binding partners, followed by probing with a BIM antibody and a secondary antibody enabling electrochemiluminescence-based quantification of MCL-1–BIM complexes (see Materials and Methods). A-1210477 caused a dose-dependent reduction in the amount of BIM co-immunoprecipitated with MCL-1, with an IC_50_ in the low-*μ*M range (Figure 2a). We observed similar dose-dependent inhibition of MCL-1–NOXA interactions using a recently described mammalian two-hybrid system (Figure 2b) that enables quantification of BCL-2 family protein–protein interactions in live cells.^11^ A-1210477 inhibited MCL-1–NOXA interactions with an IC_50_ of approximately 1 *μ*M, while having no effect on BCL-2–BIM or BCL-X_L_–BCL-X_S_ interactions measured in the same cell background (Figure 2b). Other analogs, including A-1155905, exhibited similar functional selectivity for MCL-1 (Supplementary Figure S2).

**Figure 3:**
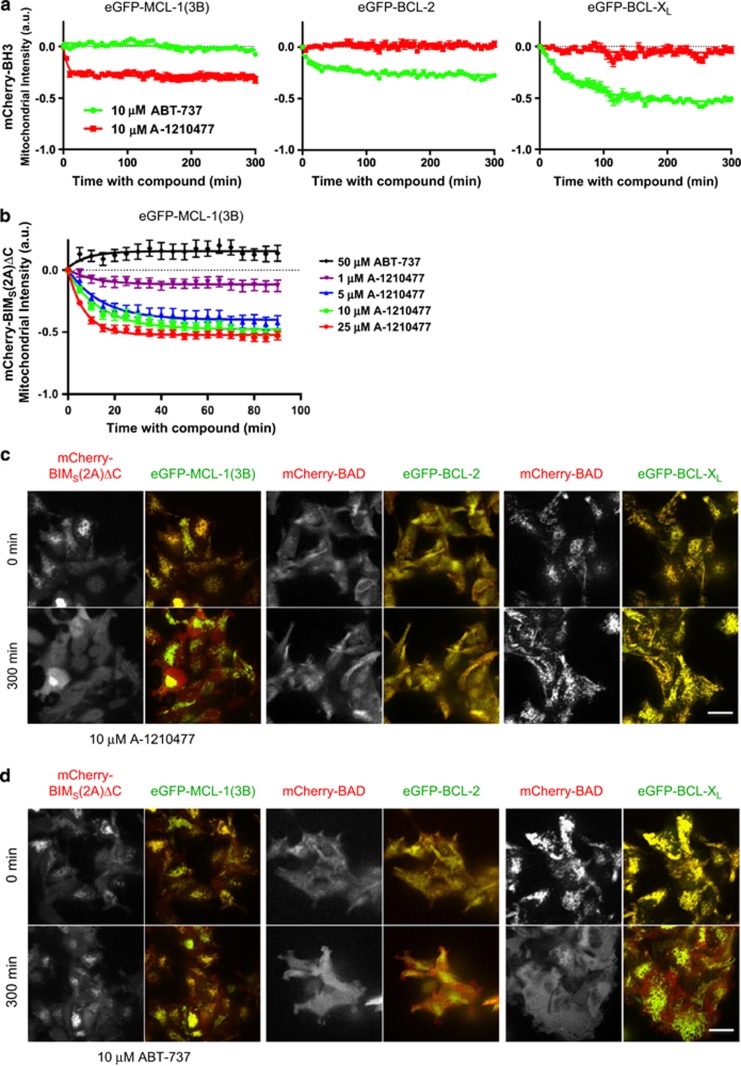
A-1210477 inhibits MCL-1-BIM complexes in live cells. (**a**) T-REx-293 cells stably expressing eGFP-MCL-1(3B) and mCherry-BIM_S_(2A)ΔC, eGFP-BCL-2 and mCherry-BAD, or eGFP-BCL-X_L_ and mCherry-BAD were treated with 10 *μ*M ABT-737, A-1210477, or dimethyl sulfoxide (DMSO) and imaged for 300 min. Compounds were added after acquisition of first image. The decrease in amount of mCherry-BH3 protein localized to the mitochondria was quantified as a function of time as described in the Materials and Methods, normalized to DMSO control, and fitted to a single exponential decay. Each data point represents the mean change in intensity calculated from 10 fields of view, with error bars indicating the S.E.M. (**b**) T-REx-293 cells stably expressing eGFP-MCL-1(3B) and mCherry-BIM_S_(2A)ΔC were treated with 50 *μ*M ABT-737 or increasing concentrations (1–25 *μ*M) A-1210477 for 100 min. The decrease in amount of mCherry-BIM_S_(2A)ΔC localized to the mitochondria was quantified as in panel (**a**). (**c**) Representative fluorescence microscopic images of T-REx-293 cells stably expressing eGFP-BCL-2 family and interacting mCherry-BH3-only proteins before and after 300 min of treatment with 10 *μ*M A-1210477 or (**d**) ABT-737. Grayscale images show the mCherry-fusion proteins only, which were pseudocolored red in the overlay with the eGFP-fusion proteins (pseudocolored green). Scale bar represents 25 *μ*m

To further rule out potential artifacts associated with lysate detergents that can influence interactions between BCL-2 family proteins,^47^ we employed a recently described real-time imaging method to assess the effects on BCL-2 family complexes in live cells.^48^ Briefly, interacting pairs of BCL-2 family proteins are expressed as fluorescent fusion proteins at stoichiometric levels from a bicistronic expression module integrated into a single site of the genome in T-REx-293 cells. Under basal conditions, both proteins localize primarily to the mitochondria, where the anti-apoptotic proteins (enhanced green fluorescent protein (eGFP)-BCL-2, eGFP-BCL-X_L_, or eGFP-MCL-1(3B)) sequester their pro-apoptotic counterparts (mCherry-BAD or mCherry-BIM_S_(2A)ΔC, the latter being a specific binder of MCL-1^48, 49^). Disruption of these complexes can be tracked by measuring the amount of mCherry-labeled BH3-only protein localized specifically to the mitochondria. If the protein complexes are disrupted by the small-molecule BH3 mimetics, mCherry signal associated with the mitochondria decreases as the BH3-only fusion protein redistributes to the cytosol. As demonstrated previously,^48^ the BCL-2/BCL-X_L_ inhibitor ABT-737 induced the redistribution of mCherry-BAD associated with BCL-2 or BCL-X_L_ (Figures 3a and d). However, ABT-737 had no effect on the distribution of BIM_S_(2A)ΔC in MCL-1(3B)-expressing cells (Figures 3a, b and d). Conversely, A-1210477 had no effect on BCL-2–BAD or BCL-X_L_–BAD complexes but induced dose-dependent redistribution of BIM_S_(2A)ΔC to the cytosol within minutes of treatment (Figures 3a–c; Supplementary Figure S3). This effect was concentration dependent and could be observed at concentrations as low as 1 *μ*M (Figure 3b). Thus data generated in four separate assays demonstrate that A-1210477 and its related analogs are able to penetrate living cells and bind to MCL-1 selectively and with sufficient affinity to compete with BH3-only proteins.

### A-1210477 and related analogs induce the hallmarks of apoptosis in MCL-1-dependent cancer cell lines

**Figure 4:**
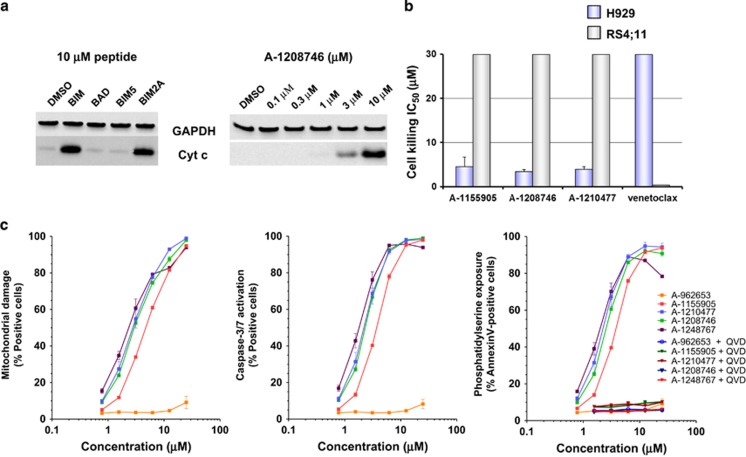
Indole-2-carboxylic acids induce the hallmarks of apoptosis in MCL-1-dependent cancer cell line H929. (**a**) Digitonin-permeabilized H929 cells were treated with 10 *μ*M BH3 peptides for 4 h, and intact H929 cells were incubated with increasing concentrations (0.1–10 *μ*M) of A-1208746 for 4 h before assessing the levels of cytochrome *c* released into the cytosol. (**b**) H929 and RS4;11 cells were incubated with increasing concentrations of MCL-1 inhibitors or the BCL-2-selective inhibitor ABT-199 (venetoclax) for 48 h before determining cell viability. Cell killing IC_50_s are plotted with each value representing the mean of three separate experiments. Error bars represent the S.E.M. (**c**) H929 cells were incubated with increasing concentrations of MCL-1 inhibitors in the presence or absence of the pan-caspase inhibitor QVD (100 *μ*M) for 4 h before assessing caspase-3/-7 activation, mitochondrial membrane polarization, and the exposure of phosphatidylserine. Data represent the mean of triplicate experiments, with error bars indicating the S.E.M.

To facilitate the assessment of A-1210477 and its related indole-2-carboxylic acid analogs, we sought to identify MCL-1-dependent cell lines that would be predicted to undergo apoptosis in response to these agents. We started by assessing multiple myeloma cell lines, which have been reported to rely on MCL-1 for survival.^25, 26^ Using BH3 profiling, a peptide-based method that has been used extensively to determine BCL-2 family dependencies,^50^ we found that the H929 cell line exhibited a clear, MCL-1-dependent profile. Cytochrome *c* was released from the mitochondria of permeabilized H929 cells treated with the BIM2A peptide, which has selective affinity for MCL-1,^49^ but not the BAD peptide, which targets BCL-2, BCL-X_L_ and BCL-W (Figure 4a). When intact H929 cells were treated with the A-1210477 analog A-1208746, cytochrome *c* was observed in cytosolic fractions within 4 h at concentrations as low as 3 *μ*M (Figure 4a), indicating that this compound can initiate apoptosis in MCL-1-dependent cells. As expected, A-1155905, A-1208746, and A-1210477 each killed H929 cells with IC_50_ values in the low-*μ*M range, while having no effect on the RS4;11 cell line (Figure 4b), which is known to rely on BCL-2 for survival.^11, 51^ These data demonstrate that these compounds selectively kill MCL-1-dependent cell lines.

To confirm that these compounds act on-mechanism, we performed a series of assays designed to assess the sequential events that occur during activation of the intrinsic apoptosis pathway. A-1155905, A-1208746, A-1210477, and A-1248767 induced the hallmarks of apoptosis in the MCL-1-dependent H929 cell line, including mitochondrial membrane depolarization, caspase-3/-7 activation, and the externalization of phosphatidylserine on the cell surface (Figure 4c). Pretreatment with the pan-caspase inhibitor QVD inhibited the externalization of phosphatidylserine, further indicating that the cell death induced by these compounds is caspase dependent and occurs by apoptosis. Furthermore, these compounds did not kill murine embryonic fibroblasts lacking the apoptosis effector proteins Bak and Bax (Supplementary Figure S4), indicating that they do not kill cells indiscriminately through a non-apoptotic mechanism.

**Figure 5:**
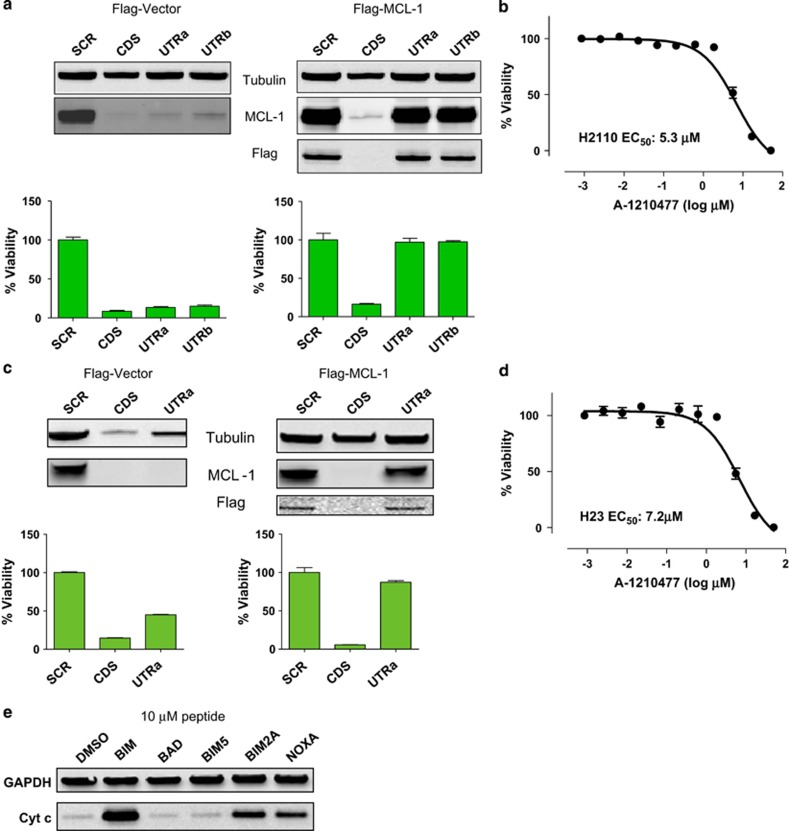
A-1210477 kills MCL-1-dependent NSCLC cancer cell lines. NSCLC cell lines (**a**) H2110 or (**c**) H23 were transfected with a construct expressing the *MCL1* CDS tagged with a Flag epitope or empty control vector. These cells were subsequently transfected with siRNAs targeting the *MCL1* CDS, the *MCL1* 3′ UTRa or UTRb, or a scrambled control sequence. Cell viability was assessed 72 h later with MCL-1 and Flag immunoblots performed in parallel. (**b**) H2110 or (**d**) H23 cells were treated with increasing concentrations of A-1210477 for 72 h before assessing cell viability. All data points represent the means of triplicate experiments, with error bars indicating the S.E.M. (**e**) Digitonin-permeabilized H23 cells were treated with 10 *μ*M BH3 peptides for 4 h before isolating cytosolic fractions, which were immunoblotted using the cytochrome *c* antibody

We next examined the effects of these molecules in a selection of solid tumor cell lines reported to rely on MCL-1 for survival, including the NSCLC cell lines H2110 and H23.^24, 29^ siRNA rescue experiments (Figures 5a and c) and BH3 profiling (Figure 5e) confirmed that these cell lines are dependent on MCL-1 for survival. Both cell lines were sensitive (cell viability IC_50_<10 *μ*M) to A-1210477 (Figures 5b and d), confirming that this compound can kill MCL-1-dependent cell lines.

### A-1210477 synergizes with navitoclax to induce apoptosis in multiple cancer cell lines

**Figure 6:**
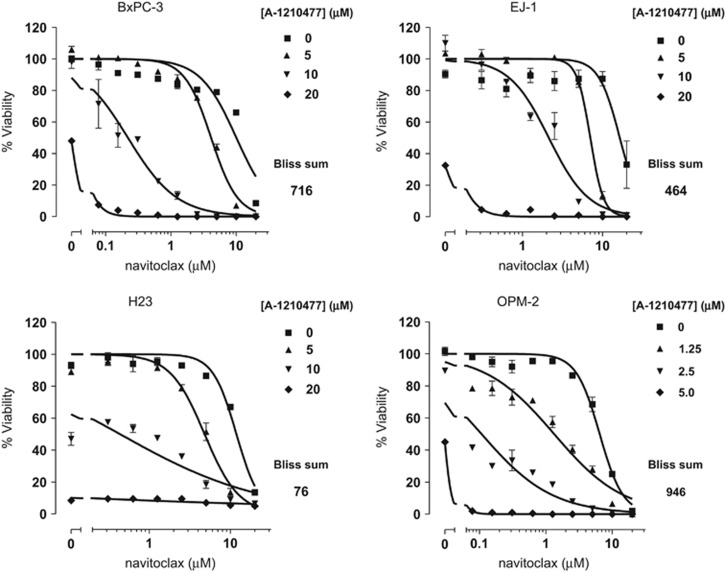
A-1210477 synergizes with navitoclax to kill hematological and solid tumor cell lines. BxPC-3, EJ-1, H23, and OPM-2 cells were treated with increasing concentrations of navitoclax in the presence or absence of increasing concentrations of A-1210477 for 48 h before assessing cell viability. All data points represent the means of triplicate experiments, with error bars indicating the S.E.M.

MCL-1 has been well characterized as a resistance factor for the BCL-2/BCL-X_L_ inhibitors ABT-737 and navitoclax. There are many examples of MCL-1 inhibition, typically via siRNA-mediated knockdown, sensitizing cancer cell lines to killing by ABT-737.^16, 17, 18, 19, 52^ We therefore tested the combination of navitoclax and A-1210477 in a panel of cancer cell lines known to depend on BCL-X_L_ and MCL-1 for survival, for example, the gastric cancer cell line EJ-1.^19, 52^ As expected, navitoclax had little or no effect on these cell lines up to 3–5 *μ*M, concentrations that will typically kill cells dependent on BCL-2 and/or BCL-X_L_ alone (Figure 6). A-1210477 substantially shifted the dose–response curve of navitoclax in BxPC-3, EJ-1, H23, and OPM-2 cells, indicating an ability to potentiate the effect of BCL-2/BCL-X_L_ inhibition. Indeed, Bliss additivity analysis indicated that these combinations were highly synergistic. These data further demonstrate that the elaborated indole-2-carboxylic acid molecules described here are indeed cell-active MCL-1 inhibitors that behave as predicted in sensitizing cancer cells to the effects of BCL-2/BCL-X_L_ inhibitors.

## Discussion

Small-molecule BH3 mimetics such as ABT-737, ABT-263 (navitoclax), and ABT-199 (venetoclax) have been used extensively to elucidate the functions of BCL-2 and BCL-X_L_ in tumor and normal cell survival. Navitoclax and the BCL-2-selective inhibitor venetoclax are also being tested in the clinic, where they are demonstrating anti-tumor activity in hematological cancers. Recently, BCL-X_L_-selective inhibitors have also been described.^12, 13, 14, 15^ MCL-1 is likely to be a resistance factor for all of these compounds and represents an attractive cancer target in its own right. However, generating direct inhibitors of MCL-1 with the properties required for on-target cell-based activity has proven to be a major challenge. Although putative MCL-1 inhibitors have been described,^35, 36, 37, 38, 39, 53, 54^ compelling evidence that they act on-mechanism has been lacking. In our experience, sub-nanomolar, and often low picomolar, affinities are required for small molecules to compete with high-affinity endogenous ligands for binding to anti-apoptotic proteins, such as BCL-2 and BCL-X_L_. Because none of the small molecules described to date have MCL-1-binding affinities in this range, it is unlikely that they are able to directly inhibit MCL-1 in a cellular context, and indeed, the mechanism of some of these molecules has been called into question.^55, 56, 57, 58^

Here we describe the first selective small-molecule inhibitors of MCL-1 with the affinity required to confer on-target cellular activity. These molecules, exemplified by A-1210477, bound MCL-1 with sub-nanomolar affinities and demonstrated an ability to disrupt endogenous MCL-1–BH3-only protein interactions in four distinct biological assays. Using mammalian two-hybrid assays and a cell imaging platform that tracks BCL-2 family interactions in real time, we found that A-1210477 could selectively disrupt MCL-1–NOXA and MCL-1–BIM2A complexes in living cells. Intriguingly, A-1210477 and indole-2-carboxylic acid analogs with similar, sub-nanomolar affinities for MCL-1 behaved akin to exogenously expressed BH3-only proteins, causing concentration-dependent elevations in MCL-1 protein that could be observed starting at sub-*μ*M concentrations. However, the less elaborated analog A-962653, which binds MCL-1 with a lower affinity (*K*_i_=130 nM) comparable to other recently described MCL-1 inhibitors, had no effect. A-1210477 also activated the intrinsic apoptosis pathway in MCL-1-dependent cancer cell lines, triggering the release of cytochrome *c* from mitochondria, caspase-3/-7 activation, and phosphatidylserine externalization within hours. Finally, these compounds selectively killed MCL-1-dependent cancer cell lines (H929, H2110 and H23) and synergized with the BCL-2/BCL-X_L_ inhibitor navitoclax to kill cell lines that rely on multiple BCL-2 family members for survival (BxPC-3, EJ-1, OPM-2).

Although other putative MCL-1 inhibitors have been shown to induce the intrinsic apoptosis pathway, this does not prove that they act directly on MCL-1. In our hands, true BH3 mimetics, such as navitoclax, venetoclax, and A-1155463, induce the hallmarks of apoptosis within 2–4 h of application to a sensitive cell population.^6, 11, 15^ Any number of indirect mechanisms can lead to apoptosis over longer time periods, and so the fact that a given molecule engages the intrinsic apoptosis pathway, often after ⩾24 h of treatment, is not sufficient to indicate that it is a direct BCL-2 family inhibitor. Furthermore, care must be taken in selecting the cell lines used to assess on-target activity. Confirmation that the cells are in fact reliant on MCL-1 for survival is essential if they are to be used as a standard for testing putative MCL-1 inhibitors. We employed a combination of biochemical (BH3 profiling) and cell/molecular biology (siRNA rescue) approaches to validate the MCL-1 dependence of cells lines such as H929, H2110, and H23, which were thereby deemed reliable to assess the effects of putative MCL-1 inhibitors. Unfortunately, validated cell lines have not always been used to assess other putative MCL-1 inhibitors. Indeed, in one case, the cell line used to assess inhibitor-mediated target inhibition and cell killing was shown to be essentially unaffected when MCL-1 protein was completely knocked down over a period of 72 h.^37^ These examples demonstrate some of the challenges and pitfalls involved with validating small-molecule inhibitors as reliable biological probes.

Workman and Collins^59^ recently reviewed the learnings of the chemical biology and drug discovery communities over several decades of synthesizing and implementing small-molecule inhibitors as probes for the dissection of biology, the validation of therapeutic targets, and the development of approved therapeutics. They described a series of ‘fitness factors', encompassing selectivity, potency, and chemical properties, to be considered when assessing the utility of a probe in a given biological context. As demonstrated here, A-1210477 and its closely related analogs fulfill these criteria: they bind MCL-1 with high selectivity relative to other BCL-2 family proteins (and also a variety of kinases and GPCRs; data not shown), exhibit sub-nanomolar affinity for MCL-1 in biochemical assays, and are able to inhibit MCL-1 directly in cells at low-micromolar concentrations. The difference between target affinity and cellular potency can be at least partially attributed to physicochemical properties. For example, the zwitterionic nature of the pharmacophore may impose limitations on passive membrane diffusion^60^ and, similar to other BCL-2 family inhibitors,^15^ A-1210477 and related analogs are highly protein bound. This is reflected by a >15-fold shift in MCL-1 affinity in the presence of 10% human serum and measured human protein binding >99%.^44^ These molecules induce all the expected hallmarks of intrinsic apoptosis in MCL-1-dependent cell lines and behave as expected in the context of cells dependent on MCL-1 for resistance to the BCL-2/BCL-X_L_ inhibitor navitoclax. Hence the preponderance of evidence presented in this study indicates that these molecules are acting on-target and are therefore reliable tools for probing MCL-1 function *in vitro*.

A key question that remains unanswered by these studies is whether normal tissues can tolerate MCL-1 inhibition at the level required for therapeutic benefit. The best predictions of potential on-target toxicities have come from Mcl-1 knockout models.^23^ Based on these studies, cardiac, hepatic, and hematological toxicities might be anticipated for small-molecule MCL-1 inhibitors.^61, 62, 63, 64, 65, 66, 67, 68^ However, because MCL-1 may perform essential functions unrelated to the sequestration of pro-apoptotic proteins,^23, 69^ it remains possible that inhibition with a small-molecule BH3 mimetic may be better tolerated than the complete loss of MCL-1 protein. It is also possible that anti-tumor efficacy might be achieved through partial inhibition of MCL-1, which could be less toxic. Indeed, the initiation of MYC-driven tumors was significantly inhibited by the knockout of a single allele of *Mcl-1*, though normal tissues remained unaffected.^30, 70^ For now, a formal proof-of-concept must await the generation of more potent MCL-1 inhibitors with improved drug-like properties. Nevertheless, A-1210477 and the other molecules described here are the first MCL-1-selective BH3 mimetics with sufficient potency to demonstrate clear, on-target cell activity. These compounds will be invaluable tools for the research community and represent another milestone in the search for targeted treatments that will benefit patients with significant unmet medical need.

## Materials and Methods

### Cell culture

All human NSCLC, small cell lung cancer, esophageal cancer and multiple myeloma cell lines were obtained from ATCC (Manassas, VA, USA) and cultured according to the vendor's specifications. T-REx-293 cells were obtained from Life Technologies (Carlsbad, CA, USA) and maintained as described previously.^48^

### Cell proliferation and viability assays

Adherent cell lines were seeded at 50 000 cells per well in 96-well plates and treated for 48 h with compounds diluted in half-log steps starting at 30 *μ*M and ending at 0.001 *μ*M. Multiple myeloma cell lines were seeded at 15 000–20 000 cells per well and treated similarly. Effects on proliferation and viability were determined using CellTiter-Glo reagent from Promega (Madison, WI, USA) according to the manufacturer's instructions. IC_50_ values were determined by non-linear regression analysis of the concentration response data.

### Cell redistribution assays

Experiments were carried out as detailed previously.^48^ Imaging was performed with a × 40 ELWD Plan Fluor objective (NA: 0.6, Nikon, Melville, NY, USA) on a Nikon Ti-E perfect focus inverted microscope equipped with a spinning disk confocal CSU-X1 (Andor, Oxford Instruments, Belfast, UK), motorized X,Y stage (Nikon), environmental chamber (OkoLab, Pozzuoli, Italy), and iXon3 897 EMCCD camera (Andor, Oxford Instruments), controlled by the NIS-Elements software (Nikon). All analysis was performed using MATLAB (version R2012b, Mathworks, Natick, MA, USA) on 16-bit grayscale images of eGFP-BCL-2 family proteins or mCherry-BH3-only proteins. The mitochondrial compartment was identified as eGFP-localized, small, isolated structures (>~4 *μ*m^2^ and <80 *μ*m^2^) above local background intensity. In contrast to Wong *et al.*,^48^ nuclear-specific areas were not observed. Therefore, the entire cell was identified with a single intensity threshold applied to the eGFP fluorescence, excluding areas identified as mitochondria. Average intensities were reported on an image-wide basis, normalized to the total area of each compartment identified. Ratio of mCherry mitochondrial intensity/cytoplasmic intensity was calculated, and data were normalized and plotted as described.^48^ Data were analyzed from 10 fields of view per condition in two separate experiments.

### Antibodies and reagents

Antibodies for MCL-1-BIM co-immunoprecipitation studies were obtained from Thermo Fisher Scientific (Waltham, MA, USA; anti-MCL-1 clone RC13) and Abcam (Cambridge, MA, USA; anti-BIM clone Y36). Secondary anti-rabbit-HRP and anti-mouse-HRP antibodies were purchased from Amersham Biosciences (Piscataway, NJ, USA). All chemicals were purchased from Sigma (St. Louis, MO, USA). Antibodies for immunoblots were obtained from Abcam (anti-NOXA clone 114C307), BD Biosciences (San Jose, CA, USA; anti-BCL-X_L_ clone 4), Sigma (anti-*β*-actin clone AC-15) and Santa Cruz (La Jolla, CA, USA; anti-MCL-1 clone S-19).

### siRNA transfections and rescue experiments

siRNAs targeting *MCL1* were obtained from Dharmacon (Lafayette, CO, USA) or Qiagen (Valencia, CA, USA). The sequences were as follows: coding sequence (CDS; Dharmacon) GCAUCGAACCAUUAGCAGAUU, untranslated region a (UTRa) (Dharmacon) CGAAGGAAGUAUCGAAUUU, and UTRb (Qiagen) CCCGCCGAAUUCAUUAAAUUA. Cells were seeded into 10 cm dishes at 1.5 × 10^6^ per dish the day before transfections. For each transfection, 12.5 *μ*l of 20 *μ*M siRNA stock were mixed with 1.4 ml Opti-MEM (Invitrogen, Carlsbad, CA, USA) for 5 min at room temperature. In all, 22.4 *μ*l of Lipofectamine 2000 (Invitrogen) were likewise mixed with 1.4 ml Opti-MEM for 5 min. The two solutions were then mixed for 30 min prior to being added to cells covered by 2.2 ml medium (final siRNA concentration of 50 nM). After 4 h of incubation at 37 °C, the transfection solution was removed and replaced by fresh medium. Cell lysates were prepared 3 days later.

### Binding affinity assays

TR-FRET-binding affinity assays were performed for BCL-2, BCL-X_L_, and MCL-1 in 4.52 mM monobasic potassium phosphate, 15.48 mM dibasic potassium phosphate, 1 mM sodium EDTA, 0.05% Pluronic F-68 detergent, 50 mM sodium chloride, and 1 mM DTT (pH 7.5) as described previously for BCL-X_L_.^6^ For MCL-1 assays, GST-tagged MCL-1 (1 nM) was mixed with 100 nM f-Bak, 1 nM Tb-labeled anti-GST antibody, and compound at room temperature (RT) for 60 min. Fluorescence was measured on an Envision plate reader (Perkin-Elmer, Waltham, MA, USA) using a 340/35 nm excitation filter and 520/525 (f-Bak) and 495/510 nm (Tb-labeled anti-GST antibody) emission filters.

### Mammalian two hybrid

HeLa cells stably expressing a GAL4-luciferase reporter were seeded into 10 cm dishes at 5 × 10^5^ cells per plate. In all, 24 *μ*g of a plasmid expressing both the ‘bait' and ‘prey' fusion proteins (GAL4DBD-BIM and VP16AD-BCL-2, GAL4DBD-BCL-X_L_ and VP16AD- BCL-X_S_, or GAL4DBD-MCL-1 and VP16AD-NOXA, respectively) were mixed with 1.5 ml OptiMEM (Invitrogen). Separately, 24 *μ*l Lipofectamine 2000 (Invitrogen) were mixed with 1.5 ml OptiMEM. Both solutions were incubated for 5 min (RT) and then mixed together for 20 min. Three milliliters of the mixture was added to cells in 12 ml culture medium lacking antibiotics, incubated at 37 °C for 24 h, and then transferred to 96-well plates at 20 000 cells per well. Compounds were added in half-log dilutions, starting from 1 *μ*M and ending at 0.0001 *μ*M for 24 h, and luciferase activity was assessed using ONE-Glo reagent (Promega). All data were plotted as the percentage of complex remaining relative to untreated controls.

### Electrochemiluminescent ELISA

After compound treatments, cells were lysed in buffer containing 1% CHAPS, 10 mM HEPES, and 150 mM NaCl with protease inhibitor cocktail (Roche, Indianapolis, IN, USA). In all, 100 *μ*g of cell lysate was added to each well of a Meso Scale Discovery (Rockville, MD, USA) 96-well plate precoated with biotinylated anti-MCL-1 antibody (clone RC13) from Thermo Fisher Scientific. MCL-1-BIM complexes were detected with rabbit anti-BIM antibody (clone Y36) from Abcam, followed by Sulfo-tagged anti-rabbit antibody (Meso Scale Discovery; cat no. R32AB-5) and Meso Scale Discovery reading buffer with surfactant (cat. no. R92TC-2) according to the manufacturer's recommendations. Each step was followed by washing three times with PBS-T (PBS+0.1% Tween-20).

### Protein extraction and immunoblot analysis

Cells were scraped into cold PBS, pelleted, and then resuspended in 100–200 *μ*l ice-cold insect cell lysis buffer supplemented with protease inhibitors (BD Biosciences Pharmingen, San Diego, CA, USA). Cells were lysed by sonication, and the debris was cleared in a microcentrifuge. In all, 40 *μ*g of lysate were loaded in each well of 6 or 10% Tris-Glycine polyacrylamide minigels (Invitrogen) for SDS-PAGE analysis. Proteins were transferred to PVDF membranes (Invitrogen), blocked for 1 h in TBS-T plus 5% (w/v) powdered blotting grade milk (Bio-Rad Laboratories, Hercules, CA, USA), and then probed overnight at 4 °C with primary antibodies at a 1 : 2000 dilution in blocking solution. Blots were developed using the enhanced chemiluminescence (ECL Plus) reagents from Amersham Biosciences.

### Mechanism of action studies

H929 cells were cultured in RPMI 1640 (Invitrogen Corp., Grand Island, NY, USA) supplemented with 10% fetal bovine serum (Invitrogen), 1% sodium pyruvate, 25 mM HEPES, 4.5 g/l glucose, and 1% penicillin/streptomycin (Sigma) and maintained in a humidified chamber at 37 °C containing 5% CO_2_. To assess the cytochrome *c* release, 1 × 10^6^ H929 cells were treated for 4 h with BH3 peptides (10 *μ*M) or compounds (half-log dilutions starting at 10.0 *μ*M and ending at 0.1 *μ*M) before generating mitochondrial and cytosolic fractions as described previously.^11^ For all other studies, H929 cells were plated in 96-well tissue culture plates at 100 000 cells/well in 100 *μ*l RPMI supplemented with 10% FBS. Caspase activation was blocked using the pan-caspase inhibitor Q-VD-OPh hydrate (QVD; Sigma). QVD (40 mM stock in DMSO) was added to the appropriate wells to a final concentration of 100 *μ*M using a HP D300 digital dispenser (Tecan, Männedorf, Switzerland), and the cells were pretreated for 1 h prior to adding the additional compounds. The MCL-1 selective compounds (10 mM stocks in DMSO) were added using a HP D300 digital dispenser, and the cells were treated for an additional 4 h. Following treatment, the hallmarks of apoptosis (activation of caspase-3/-7, mitochondrial membrane depolarization, and phosphatidylserine externalization) were measured using the IntelliCyt (Albuquerque, NM, USA) MultiCyt 4-Plex Apoptosis Screening Kit according to the manufacturer's instructions and read by flow cytometry using a HTFC screening system (IntelliCyt). Briefly, 20 *μ*l of the treated cells were transferred to a 96-well V-bottom plate containing a 20 *μ*l cocktail consisting of a proprietary combination of dyes and incubated for 1 h at 37 °C before reading. Data were expressed as the percentage of singlet cells. For the remaining 80 *μ*l of treated cells, viability was determined using CellTiter-Glo reagent (Promega).

## Abbreviations

BH3: BCL-2 homology 3
CDS: coding sequence
eGFP: enhanced green fluorescent protein
ELISA: enzyme-linked immunosorbent assay
NSCLC: non-small cell lung cancer
TR-FRET: time-resolved fluorescence resonance energy transfer
UTR: untranslated region
