# Supplementary material for: Potent and selective small-molecule MCL-1 inhibitors demonstrate on-target cancer cell killing activity as single agents and in combination with ABT-263 (navitoclax)
Source: Cell Death Dis. 2015 Jan 15;6(1):e1590–. doi: 10.1038/cddis.2014.561 (PMC4669759; doi:10.1038/cddis.2014.561)
Supplement: Supplementary Information [file cddis2014561x1.ppt]

## Slide 1
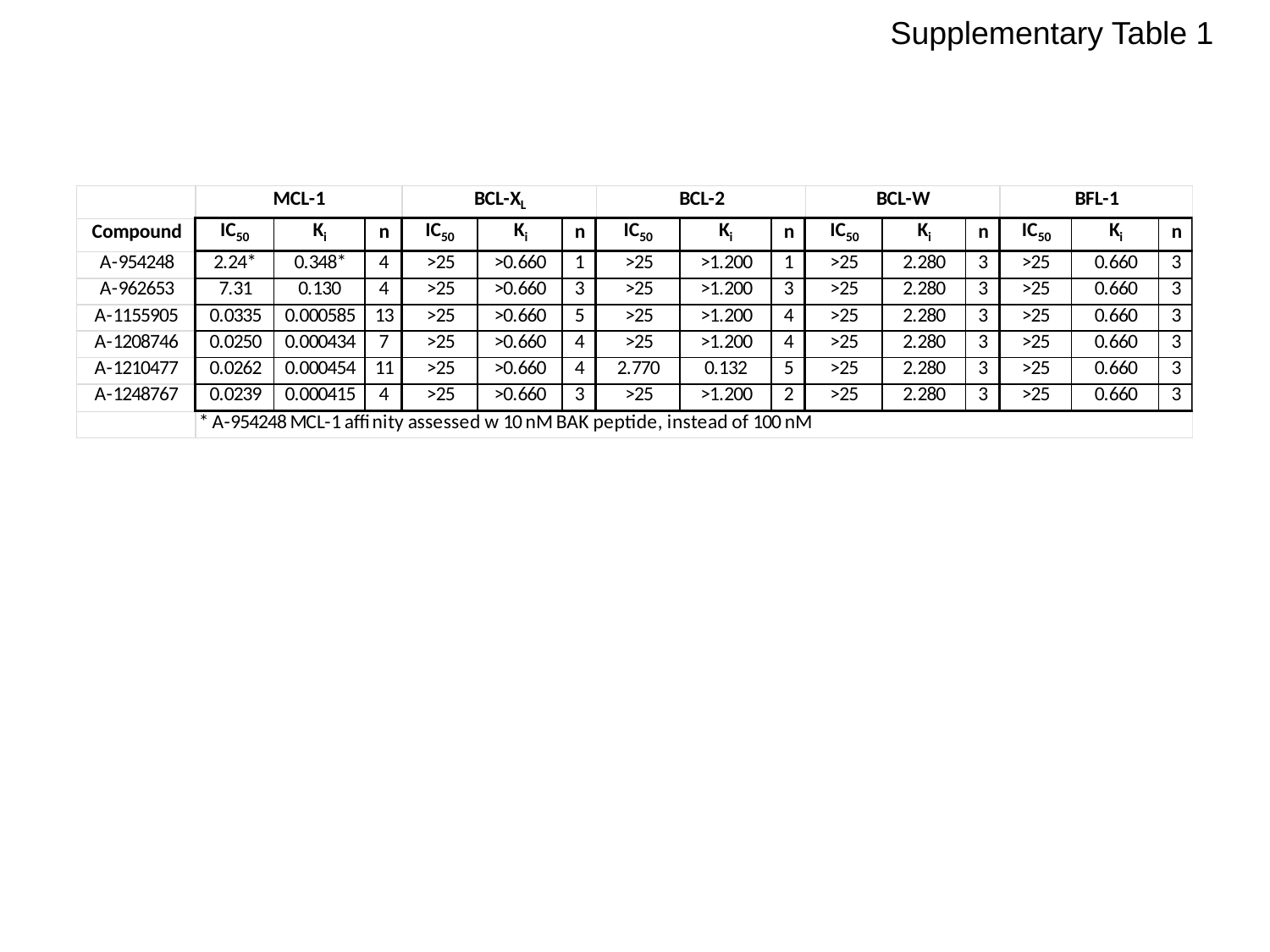

Supplementary Table 1

## Slide 2
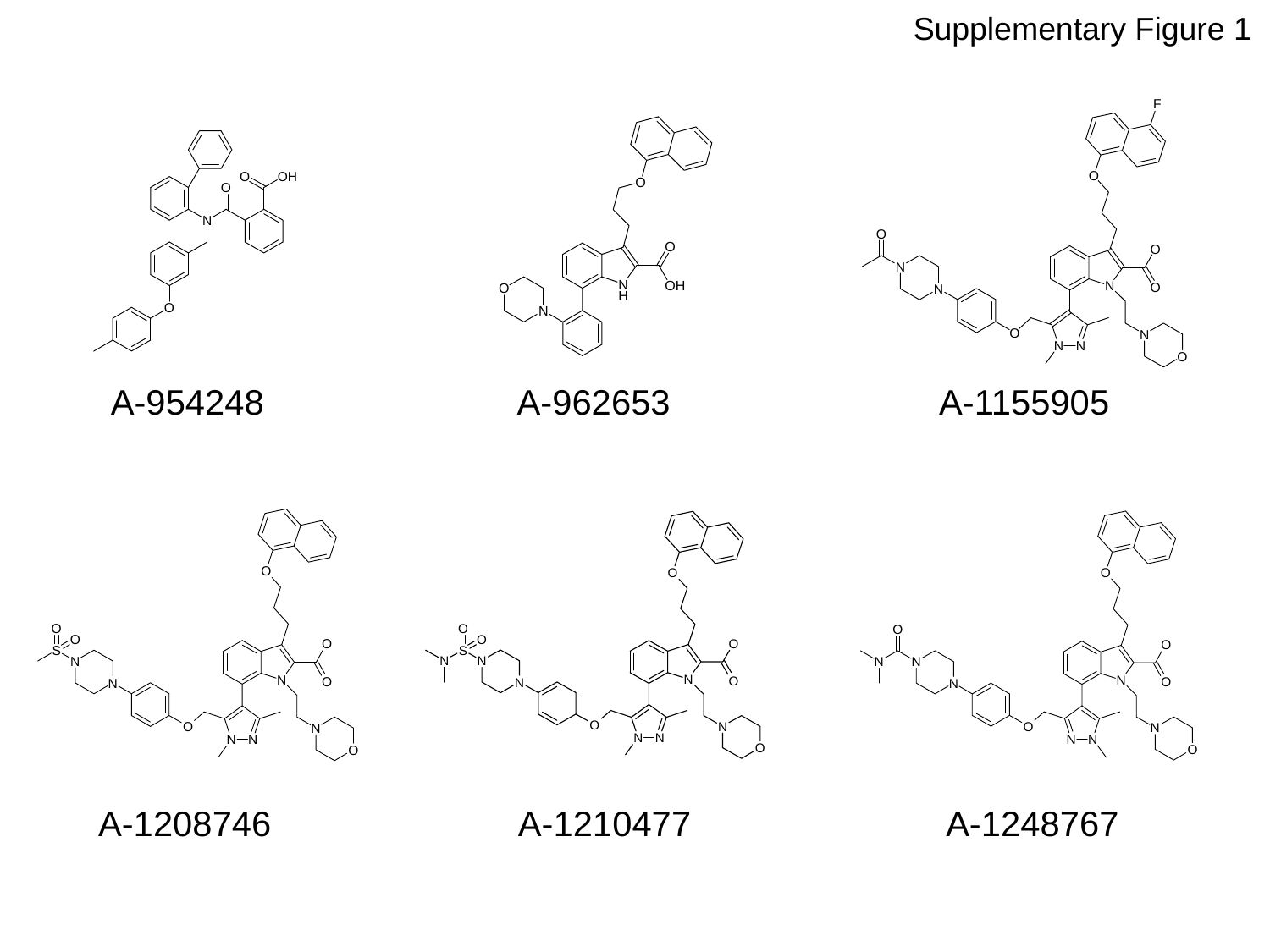

Supplementary Figure 1
A-954248
A-962653
A-1155905
A-1208746
A-1210477
A-1248767

## Slide 3
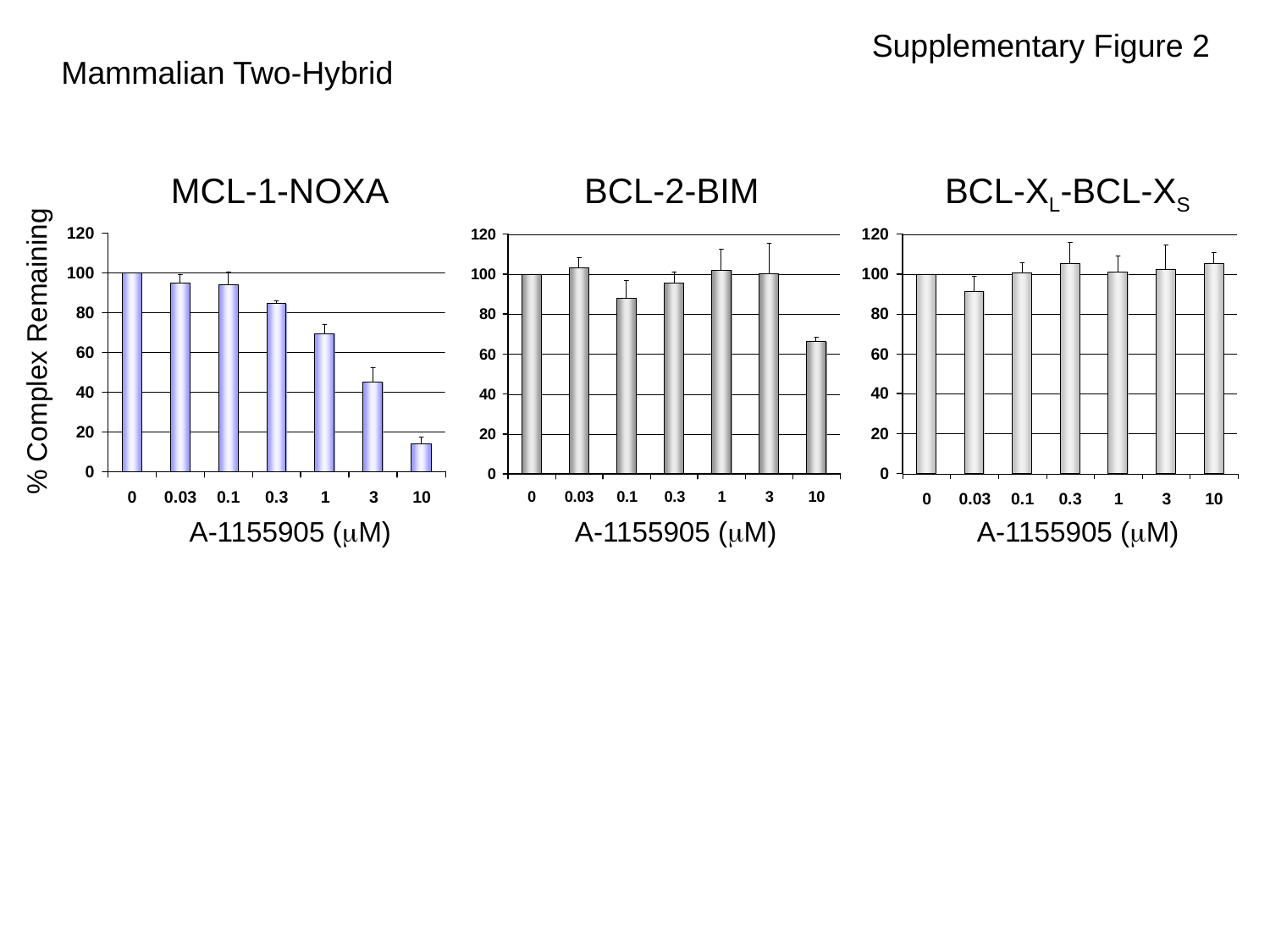

Supplementary Figure 2
Mammalian Two-Hybrid
MCL-1-NOXA
BCL-2-BIM
BCL-XL-BCL-XS
% Complex Remaining
A-1155905 (μM)
A-1155905 (μM)
A-1155905 (μM)

## Slide 4
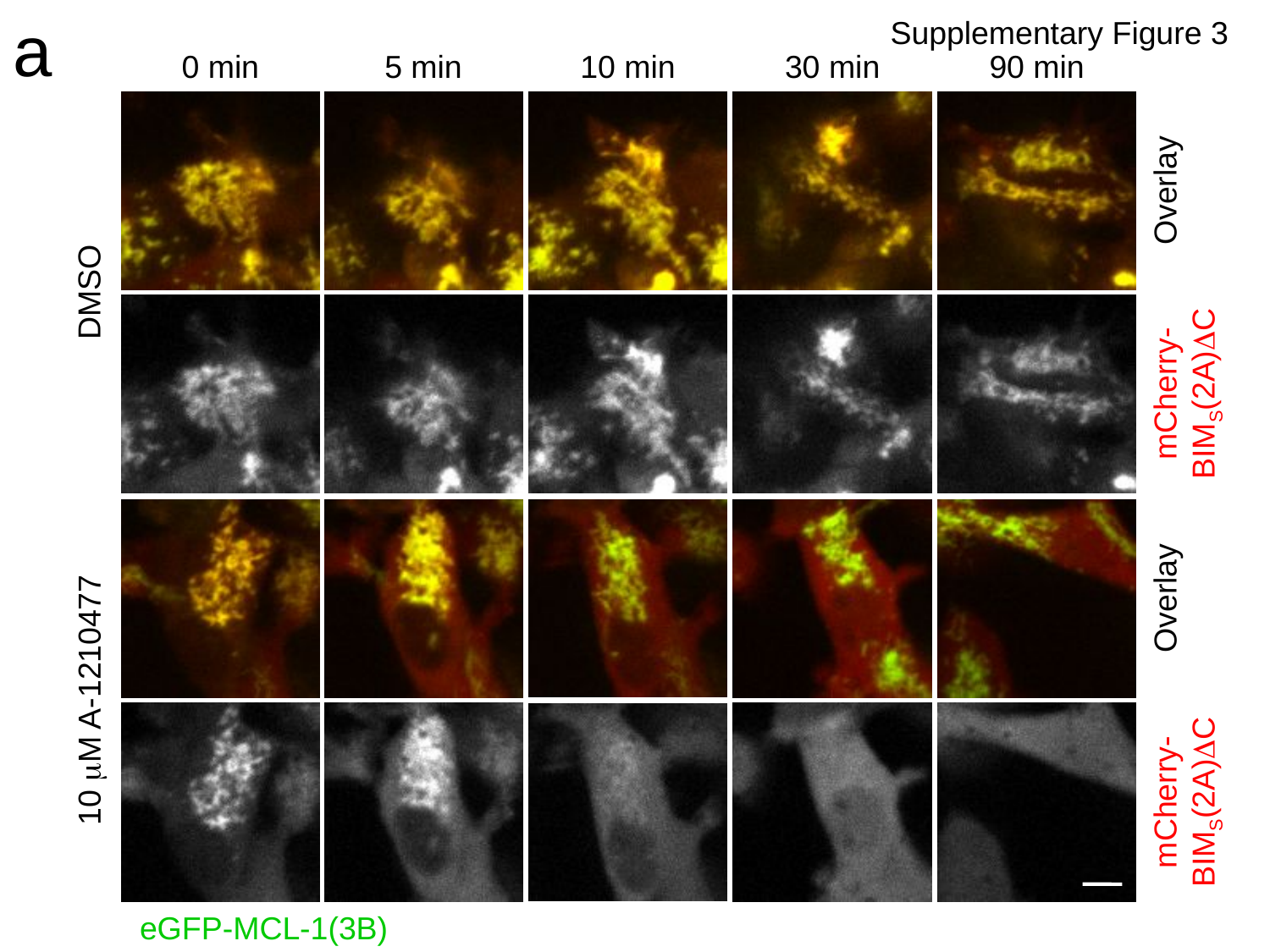

a
Supplementary Figure 3
0 min
5 min
10 min
30 min
90 min
Overlay
DMSO
mCherry-
BIMS(2A)ΔC
Overlay
10 μM A-1210477
mCherry-
BIMS(2A)ΔC
eGFP-MCL-1(3B)

## Slide 5
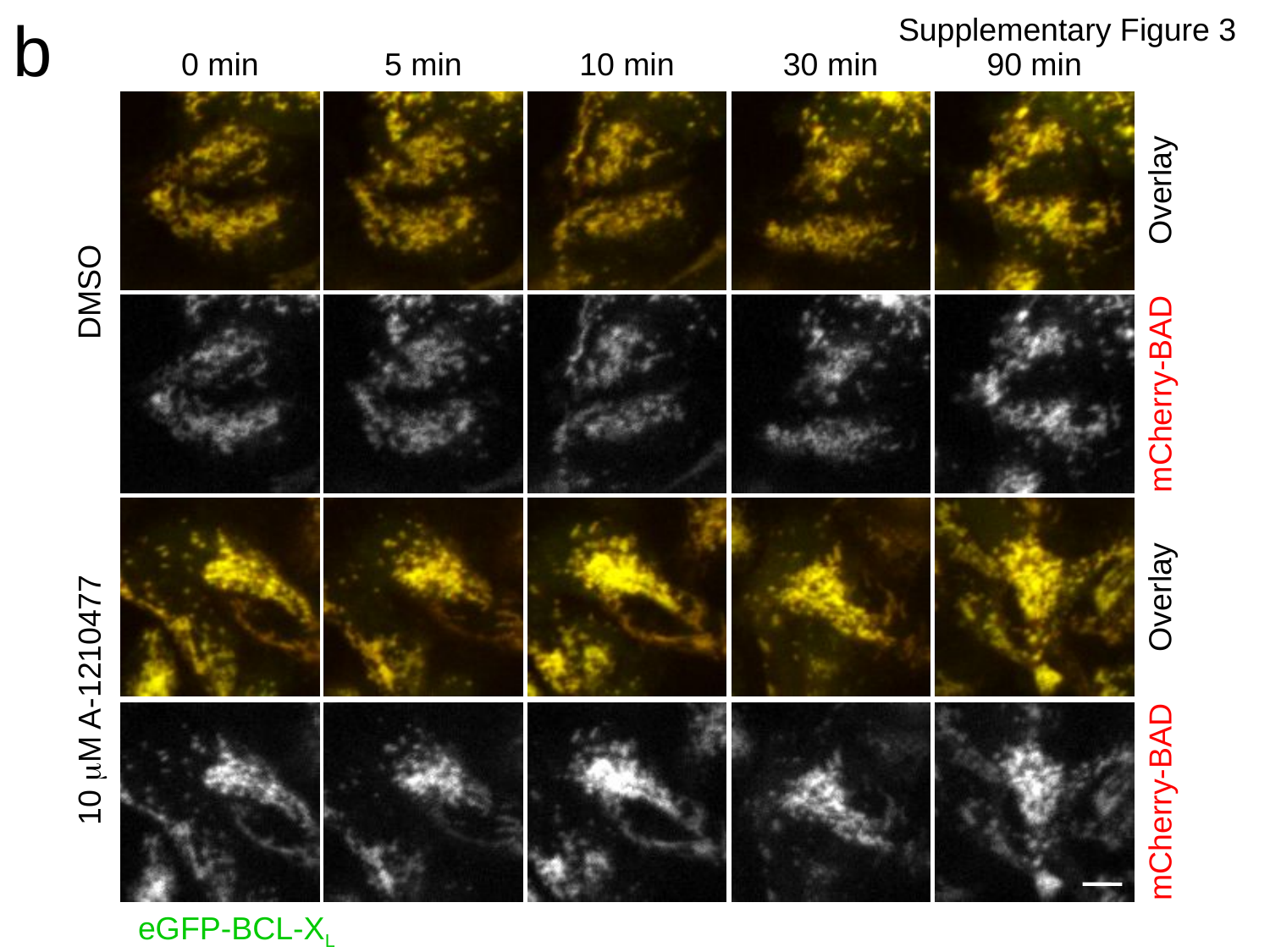

b
Supplementary Figure 3
0 min
5 min
10 min
30 min
90 min
Overlay
DMSO
mCherry-BAD
Overlay
10 μM A-1210477
mCherry-BAD
eGFP-BCL-XL

## Slide 6
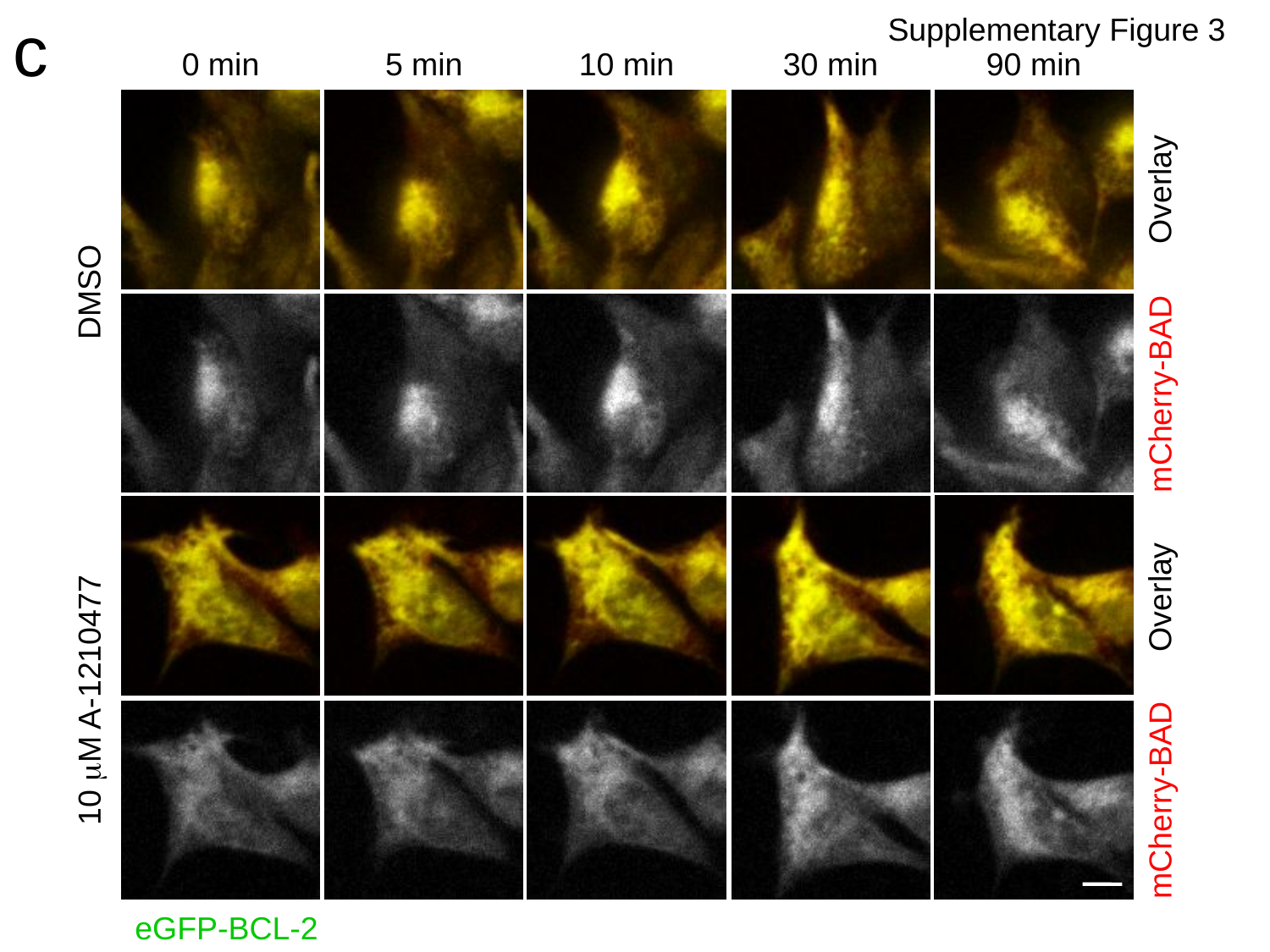

c
Supplementary Figure 3
0 min
5 min
10 min
30 min
90 min
Overlay
DMSO
mCherry-BAD
Overlay
10 μM A-1210477
mCherry-BAD
eGFP-BCL-2

## Slide 7
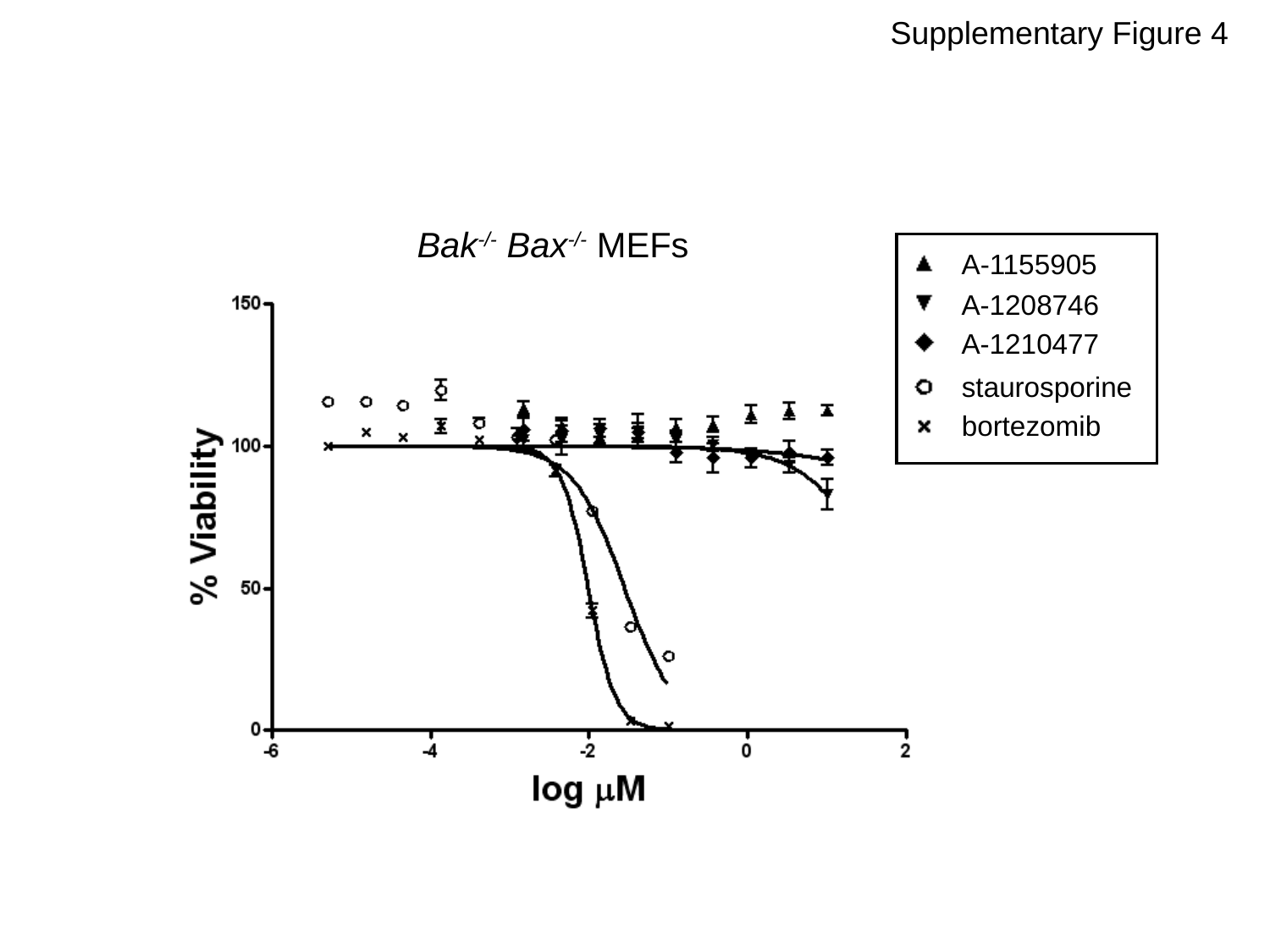

Supplementary Figure 4
Bak-/- Bax-/- MEFs
A-1155905
A-1208746
A-1210477
staurosporine
bortezomib
