# Supplementary material for: Potent and selective small-molecule MCL-1 inhibitors demonstrate on-target cancer cell killing activity as single agents and in combination with ABT-263 (navitoclax)
Source: Cell Death Dis. 2015 Jan 15;6(1):e1590–. doi: 10.1038/cddis.2014.561 (PMC4669759; doi:10.1038/cddis.2014.561)
Supplement: Supplementary Information [file cddis2014561x2.doc]

**Supplementary Figure Legends**

**Supplementary Table 1.** **Small-molecules with selective binding affinity for MCL-1.** IC_50_ values and inhibition constants (K_i_) calculated in BCL-2 family TR-FRET assays are listed in µM units for each compound used in this study. The number of independent replicates (n) carried out for each binding assay is listed.

**Supplementary Figure 1. Indole-2-carboxylic acid-based compounds are selective MCL-1 inhibitors.** The chemical structures of the indole-2-carboxylic acid compounds utilized in this study are depicted.

**Supplementary Figure 2. MCL-1 Inhibitor A-1155905 Disrupts MCL-1-BIM Complexes.** Quantitative measurement of protein-protein interactions using a luciferase-based mammalian two-hybrid assay. HeLa cells stably transfected with a GAL4-driven luciferase reporter and expressing paired GAL4DBD-MCL-1:VP16AD-NOXA, VP16AD-BCL-2:GAL4DBD-BIM or GAL4DBD-BCL-X_L_:VP16AD-BCL-X_S_ fusion proteins were incubated with increasing concentrations of A-1155905 for 24 hours. All data represent the means of triplicate experiments with error bars indicating the standard deviations.

**Supplementary Figure 3. A-1210477 Disrupts MCL-1-BIM Complexes in Live Cells.** Time-lapse images of representative T-REx-293 cells stably expressing A) eGFP-MCL-1 (3B) and mCherry-BIM_S_(2A)ΔC, B) eGFP-BCL-X_L_ and mCherry-BAD, or C) eGFP-BCL-2 and mCherry-BAD treated with 10 µM A-1210477 or DMSO control following first image acquisition. Scale bar represents 10 µm.

**Supplementary Figure 4. MCL-1 Inhibitors do not Kill *Bak^-/-^ Bax^-/-^*MEFs.** Murine embryonic fibroblasts (MEFs) lacking Bak and Bax were treated with increasing concentrations of A-1155905, A-1208746, A-1210477, staurosporine or bortezomib for 48 h before assessing cell viability. Data represent the mean of triplicate experiments and error bars indicate the standard error of the mean.
